# Vitamins (A&D) and Isoprenoid (Chenodeoxycholic acid) molecules are accompanied by Th1 immunostimulatory response and therapeutic cure *in vivo*: possible antileishmanial drugs

**DOI:** 10.1038/s41598-019-44630-4

**Published:** 2019-06-12

**Authors:** Venkateswara Reddy Gogulamudi, Mohan Lal Dubey, Deepak Kaul, Donfack Jean Hubert, Ramesh Kandimalla, Rakesh Sehgal

**Affiliations:** 1 Department of Medical Parasitology, Postgraduate Institute of Medical Education and Research, 160012 Chandigarh, India; 2 Department of Biochemistry, Postgraduate Institute of Medical Education and Research, 160012 Chandigarh, India; 3 Department of Experimental Medicine and Biotechnology, Postgraduate Institute of Medical Education and Research, 160012 Chandigarh, India; 4 Department of Pharmaceutical Sciences, University of Dschang, P.O. Box 96, Dschang, Cameroon

**Keywords:** Target identification, Infectious diseases

## Abstract

Investigation of immune modulatory anti-leishmanial molecules is now being strongly encouraged to overcome the immunosuppression manifested during visceral leishmaniasis (VL), resistance, toxicity and high cost associated with conventional therapeutics. In the present study, we explored the protective efficacy of vitamin D_3_, retinoic acid and isoprenoid chenodeoxycholic acid (CDCA) combinations against *L*. *donovani* infected BALB/c mice. We also probed the immune modulatory response (Th1 & Th2 cytokines) and infection dynamics following experimental infections with drug treated animals. Our results indicate that Vit.D_3_/RA and CDCA/RA combination treatment led to significant inhibition of parasite load on days 21 and 28 post treatment. Furthermore, there was a marked inhibition of Th2 type immune responses in IL-4, IL-5 and polarization of Th1 biased immunity along with upregulation of IL-1, IFN-γ, and TNF-α levels on day 28 post treatment. In addition, mice treated with Vit.D_3_/RA and CDCA/RA demonstrates here that splenic histological recovery against the virulent challenge of *L*. *donovani* by day 28 was comparable to control group. The conclusions derived from this study suggests that a combination of vitamin A, D_3_ and isoprenoids may have a potential immunomodulatory therapeutic role against leishmaniasis.

## Introduction

Visceral Leishmaniasis (VL), known as kala-azar, caused by a protozoan parasite, becomes a fatal disease if left untreated. It is among the most important neglected tropical diseases in the world. The illness ranges from self-healing to very serious, and clinical manifestations include VL. The World Health organization estimated that 1.3 million new cases and 30,000 deaths occur annually^1^. In addition, HIV co-infection with Leishmania is becoming more and more problematic in industrialized, developing, non-endemic countries. Leishmania has evolved a variety of remarkable strategies to invade the mammalian protection system by both innate and acquired immune effector mechanisms. VL affects mainly internal organs like the liver, spleen and bone marrow. Progression of VL is categorized by profound immunosuppression in experimental animals and humans^2,3^, which results in acute parasite load. Unfortunately, there are few drugs available for the treatment of leishmaniasis. Therapeutic armamentarium is currently plagued with several restrictions as the available drugs are potentially toxic, effective only parenterally, expensive and need to be administered for extended periods. Additionally, in the absence of a vaccine, the treatment options for VL are limited and far from satisfactory. Further, multi-drug resistant strains of leishmaniasis are on the rise^4^, and HIV/VL coinfected patients are another possible source for the emergence of drug resistance^5^. Thus, there is an urgent need to develop new, safer anti-leishmanial drugs to supplement those in present use. Apart from having potent leishmanicidal activity, the ideal drug should be affordable, relatively free of toxicity and be administered orally, in combination, to avoid resistance issues. Attempts to develop newer synthetic compounds have met with limited success.

The initial host-parasite interactions represent a crucial event in the establishment of primary infection. The outcome of the disease depends on the parasite species, the host genetics and immune activity to infect. The early innate immune reactions by the host, including macrophage-parasite interaction, are crucial in determining the persistence of the parasites^6,7^. Therefore, an ideal drug should boost the host immune system inducing specific memory for cell-mediated immune responses, which concurrently cause parasite death can certainly benefit the host against leishmanisis. This will result in effectively leading the host immunse system to reduce treatment failure and reinfection. However, the relationship between anti-leishmanial drug therapy and host immunity is an area which has received little attention. Research efforts on leishmaniasis over recent years have been focused on the escape mechanisms of host immune defense, prevention, and treatment, which, among other advances, have provided us with new insights into the complex network of parasite-cytokine interaction^8^. Recently, we have shown in an *in vitro* study that tryptophan aspartate-containing coat (TACO) protein, a coat protein on phagosomes, facilitates the entry, survival and downregulation of the gene has a marked role in parasite clearance^9^. However, the possible involvement of TACO gene downregulation with Vitamin D_3_/Retinoic acid and Chenodeoxycholic acid/Retinoic acid combination of molecules on parasite replication and immune response is not known.

In recent years, significant progress has been made in the identification of drug targets which could induce a protective immune response against Leishmania infection. The role of vitamin molecules as antidotes against several infectious diseases has been well documented^10–12^. Vitamins are organic components in food that are needed in very small amounts for growth and for maintaining good health. Vitamins and isoprenoids are simple organic compound molecules and have become a focus of modern medicine as they have been employed for many intracellular pathogens. Vitamin D_3_ recent research has been demonstrated to have therapeutic effects on various organs and tissues; more precisely, its immunoregulatory effect has been studied in the development and resolution of various infectious diseases^13,14^. However, its exact role in leishmaniasis has not been established. In addition, another vitamin, Retinoic acid (Vit. A) has been shown to prevent various infectious diseases such as diarrhea and respiratory diseases. To date, there are few *in-vivo* studies investigating the effect of vitamin A in visceral leishmaniasis (VL)^15^. In the case of tuberculosis, vitamins A and D have shown to work synergistically to induce a decrease in TACO gene transcription^16^. Further, they also demonstrated the therapeutic role of another molecule Chenodeoxycholic acid (CDCA) in combination with vitamin A regulation of *Mycobacterium tuberculosis* infection^12^. In brief, CDCA (an isoprenoid molecule) is a bile acid that prevents the formation of the gallstone by inhibiting the cholesterol absorption (in the intestine) and production (in the liver). However, the utility of these molecules as antileishmanial agents has not been studied in visceral leishmaniasis. In addition, combination drug treatments have yielded positive results and may be a therapeutic solution to delay or prevent the development of resistance, increase efficacy, or limit the course of treatment time^17,18^. We demonstrated here the efficacy of vitamins, the role of the isoprenoid molecule as antileishmanial agents, and illustrated the mechanism to sweep over the essential events in relation to parasite clearance and immune activation on experimental model of VL.

## Materials and Methods

### Leishmania donovani strain

The standard strain of *Leishmania donovani* MHOM/IN/80/DD8 maintained in the Department of Parasitology, PGIMER, Chandigarh, by serial *in vitro* culture was used in this study by passing through BALB/c mice for maintenance of virulence. In brief, DD8 strain of *L*. *donovani* was passaged in BALB/c mice every 3–4 weeks to revive the virulence of the strain. After sacrifice, the infected spleen of the mice was removed aseptically, hand homogenized using a mesh under sterile conditions and suspended in M199 with 30% FBS. This suspension was incubated at 22 °C for 48 to 72 hours. Homogenized infected spleen tissue released amastigotes conversation to promastigotes were checked under a microscope and counted. The suspension was centrifuged at 1000 rpm for 10 min at 4 °C to remove splenic debris and the promastigotes were pelleted down at 5000 rpm for 15 min at 4 °C. The pellets were resuspended in PBS (pH 7.4) at a concentration of 10^8^ cells/ml. 100 μl of freshly transformed promastigotes (10^8^ cells/ml) were injected into the tail vein of 2–4 weeks old mice for further passage.

### Animal model

Inbred BALB/c mice, 6–8 weeks old weighing 25–28 grams(gm), were used throughout the study. These were obtained from the Central Animal Facility, Postgraduate Institute of Medical Education and Research (PGIMER), Chandigarh. The handling and maintenance of animals in all experiments were performed according to the institutional guidelines. Ethical approval for this study was granted by the Ethical Committee of the PGIMER, Chandigarh, India.

### Evaluation of therapeutic efficacy of Vit.D_3_/RA and CDCA/RA treatment in *L*. *donovani* infected BALB/c mouse model

For infection, 100 µl (10^−7^ promastigotes) suspension of stationary phase promastigotes were administered intravenously (IV) to each mouse. The establishment of infection was assessed by sacrificing 3–4 animals and measuring the parasite loads in their livers and spleens on different post-infection (PI) days. The IV inoculation of *L*. *donovani* promastigote to the BALB/c mice model results in parasite load in the liver and spleen, which peaks at 18–21 days post-infection. BALB/c mice PI were divided into three groups on day 21. Each group consisted 6 animals, for each test dose. Animals in Group I were treated as a control and given PBS only. Group II and Group III were treated orally with the combination of Vitamin D_3_/Retinoic acid (Vit.D_3_/RA) and Chenodeoxycholic acid/Retinoic acid (CDCA/RA) respectively by the oral route. The Vit.D_3_/RA and CDCA/RA treatments were started on day 21 PI and daily doses were continued for 4 weeks. All the titer values and concentrates of the molecules were extrapolated based on our previous findings^9^. The doses used were 384 ng/30 ng/mice for Vit.D_3_/RA and 78 ng/30 ng/mice for CDCA/RA. Animals were sacrificed on days 7, 14, 21 and 28 after the treatment (Table 1). Weights of spleen and liver were taken and impression smears were prepared and stained with Giemsa to assess the parasite load. Blood was collected and serum samples were stored at −20 °C for cytokine study.

**Table 1:** Infection and treatment schedule in BALB/c mice.

Infection development treatment & sacrificed	Treatment Group – I (Control)	Treatment Group – II Vit. D_3_/RA	Treatment Group – III CDCA/RA
Infection development for 21 days	2 × 10^7^ promastigotes (i.v.)	2 × 10^7^ promastigotes (i.v.)	2 × 10^7^ promastigotes (i.v.)
Day 7 (P.I.)	PBS (100 µl)	Oral Vit. D_3_/RA 384 ng/30 ng/kg	Oral CDCA/RA 78 ng /30 ng/kg
Day 14 (P.I.)	PBS (100 µl)	Oral Vit. D_3_/RA 384 ng/30 ng/kg	Oral CDCA/RA 78 ng /30 ng/kg
Day 21 (P.I.)	PBS (100 µl)	Oral Vit. D_3_/RA 384 ng/30 ng/kg	Oral CDCA/RA 78 ng /30 ng/kg
Day 28 (P.I.)	PBS (100 µl)	Oral Vit. D_3_/RA 384 ng /30 ng/kg	Oral CDCA/RA 78 ng /30 ng/kg

### Parasite load determination

Parasitological parameters were studied in each animal in treatment and control groups to estimate the drug molecules efficacy. After treatment, liver and spleens were separated and weights were taken. For the determination of parasite burden, the Leishmania-Donovani Units (LDU) were calculated as described by Stauber *et al*.^19^. In brief, a small tissue wedge was cut from spleen and liver lobe placed in RPMI. Repeated imprints were made on clean glass microscopic slides from the cut end after applying filter paper. Imprints were air dried, fixed in methanol and stained with Giemsa stain (1:10 dilution in PBS, pH 7.2) for 30 min. Amastigotes were counted in impression smears using an oil immersion objective. The LDU was calculated as follows:

$$\frac{{\rm{Number}}\,{\rm{of}}\,{\rm{amastigotes}}}{1000\,{\rm{Number}}\,{\rm{of}}\,{\rm{organ}}\,{\rm{nuclei}}}\times {\rm{Weight}}\,{\rm{of}}\,{\rm{organ}}\,{\rm{in}}\,{\rm{milligrams}}$$

The results were evaluated by comparing the parasite load of test and control group of mice.

### Detection of Th1/Th2 cytokines

Whole blood was collected in sterile tubes at the post-treatment (PT) stage and was allowed to coagulate for 2 to 3 h at 4 °C prior to centrifugation. Serum was preserved at −20 °C until cytokine measurement was performed using the cytometric bead array Mouse Th1/Th2 cytokine kit (BD Biosciences, USA) protocol. In brief, Mouse Th1/Th2 cytokine standards were reconstituted in 2 ml assay diluent and equilibrated for at least 15 min before making dilutions. The vial was agitated thoroughly and the FACS tubes were labeled in Top Standard as follows: 1:2, 1:4, 1:8, 1:16, 1:32, 1:64, 1:128 and 1:256, then 300 µl of assay diluents was transferred to each of the labeled dilution tubes. From the top standard, 300 µl was transferred to the 1:2 dilution tube and mixed thoroughly. The serial dilutions were made by transferring and thoroughly mixing 300 µl 1:2 tube to the 1:4 tube and so on. The assay diluent served as a negative control. From each mouse 10 µl of serum cytokine captured in bead suspension was mixed in a centrifuge tube and 50 µl of mixed beads were transferred to each assay tube. Then 50 µl/test PE detection reagent was added. Standard dilutions and test samples (serum) were added to the appropriate sample tubes (50 µl/tube). After 2 h of incubation at room temperature in the dark, the samples were washed with 1 ml of wash buffer and centrifuged at 200 g for 5 min. Finally, 300 µl of wash buffer was added to each assay tube, vortexed properly and the samples were analyzed by FACS Calibur (Becton Dickinson, CA, USA). The quantity (pg/ml) of respective cytokine was calculated using FCAP software. Standard curves were derived from the cytokine standards supplied with the kit.

### Histological responses

The spleen and liver tissues were fixed in 10% formalin saline dehydrated in ethanol and embedded in paraffin. Paraffin blocks of tissue sections were cut and stained with haematoxylin and eosin (H&E). To determine the degree of cellular response, each infected field focus was analyzed under the microscope (Olympus BX51, Japan). Images were obtained with a CCD camera (Olympus, model no. E330-ADU1. 2X, Japan).

### Statistical analysis

The statistical significance of the differences between the various groups and within the groups was determined by analysis of variance (ANOVA). All data were expressed as the mean values ± S.D. of triplicate samples. Differences were considered statistically significant for p < 0.05.

## Results

### Course of Infection and effect of drug treatment on parasite load in *L. donovani* infected BALB/c mice

#### Relative weight of spleen and liver

While studying the course of *L*. *donovani* infection in BALB/c mice, it was observed that parasite load (LDU) increased significantly up to day 21 PI, causing a weight increase in spleen and liver. Therefore, on day 22 PI, oral treatment with the molecules Vit.D_3_/RA and CDCA/RA was started. The combination treatment started on day 22, and daily doses were continued for 28 days. During the treatment, the spleen weight, liver weight and parasite load (LDU) were recorded every week on day 7, 14, 21 and 28 post-treatment.

**Figure 1:**
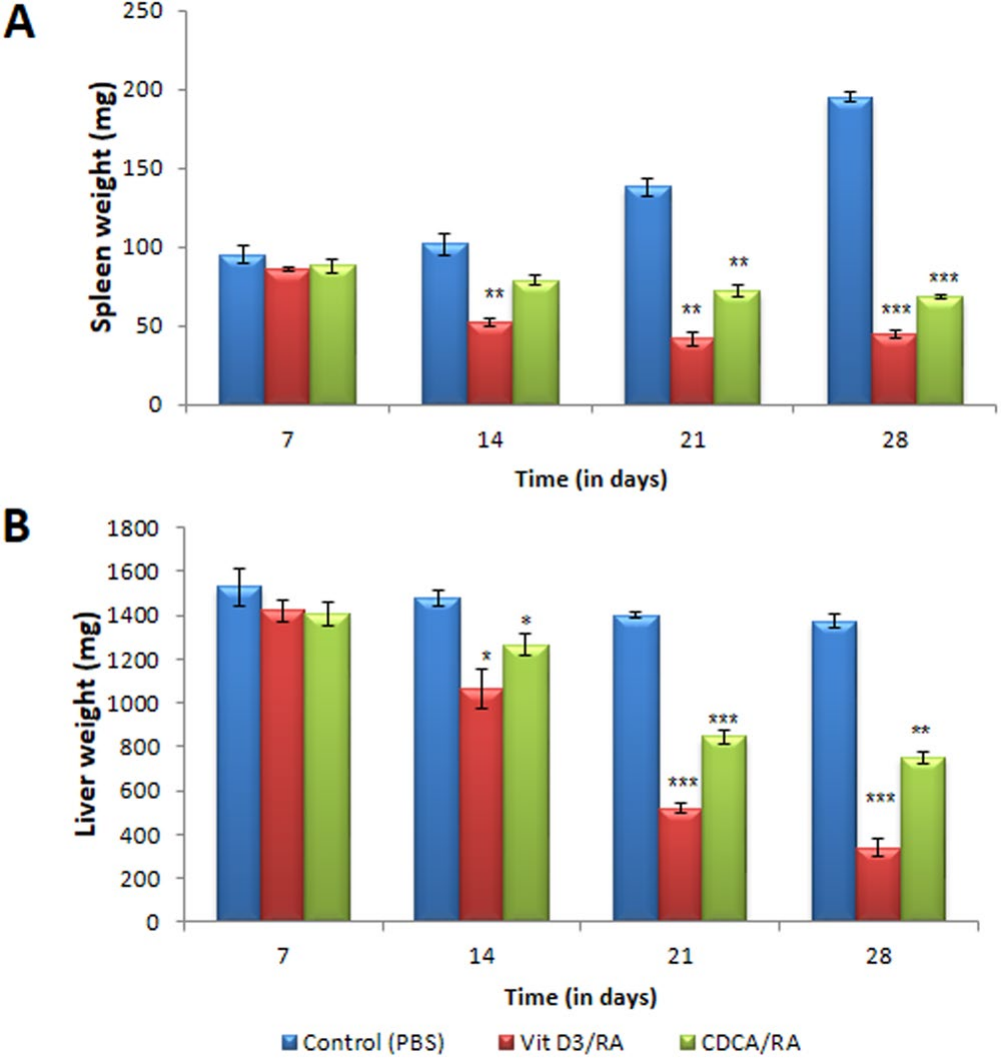
Comparison of weights of spleen & liver with Vit.D_3_/RA & CDCA/RA in course of BALB/c mice infection with *L*. *donovani*. All the treated and control groups mice were sacrificed different time intervals. (**A**) Spleen weight (**B**) Liver weight. The values represent the mean ± SD difference between the infected and control of experiments. *P < 0.05,**P < 0.005, ***P < 0.0001, n = 6.

The mean spleen weight in vehicle (PBS) treated control mice on day 7 PT was 95 ± 5.2 mg and 102 ± 6.8 mg on day 14 PI. The spleen weight increased further to 138 ± 5.4 mg on day 21 and 195 ± 3.5 mg on day 28 (Fig. 1A), whereas mice treated with Vit.D_3_/RA showed a significant decrease in spleen weight. Similarly, spleen size was decreased from 85 ± 1.5 mg (10%) on day 7 PT, 52.4 ± 2.6 (30%) on day 14, 45 ± 2.2 mg (70%) on day 21, and maximum reduction on day 28 PT 41.6 ± 4.2 mg (77%) was observed (Fig. 1A). In the group of CDCA/RA treatment, a similar decrease in spleen weight was observed, from 88 ± 4.2 mg (7%) on day 7, 79.2 ± 2.5 mg (5%) on day 14, 72.4 ± 4.2 mg (47%) on day 21 and 68.6 ± 2.2 (64%) mg on day 28 P.T (Fig. 1A). Maximum reduction was seen in treating groups, i.e., Vit.D_3_/RA (77%) (*p* = 0.0001) and CDCA/RA (64%) (*p* = 0.0001) when compared to the vehicle treated controls (Fig. 1A).

Mean weight of liver in the control group on day 7 PT was 1528 ± 85.5 mg. This further declined to 1478 ± 38.3 mg on day 14, 1400 ± 15.6 on day 21 and 1374 ± 31.3 mg on day 28 PT respectively (Fig. 1B). In the Vit.D_3_/RA treated group there was a significant decline in liver weight from 1420 ± 45.4 (7%) on day 7 to 1065 ± 88.2 mg (27%) on day 14, 520 ± 24.5 mg (62%) on day 21 and 337 ± 40.5 mg (75%) on day 28 PT (Fig. 1B). Further, treatment with CDCA/RA showed a similar decrease in liver weight from 1410 ± 54.1 mg (7%) on day 7 to 1264 ± 50.2 mg (14%) on day 14, 845 ± 35.0 mg (46%) on day 21 and 754 ± 27.2 mg (40%) on day 28 P.T (Fig. 1B). Both groups (drug treated) showed a significant decrease, i.e. in liver weight (Vit.D_3_/RA (75%) (*p* = 0.001) and CDCA/RA (46%) (*p* = 0.001) compared to the vehicle treated control group (Fig. 1B).

### Parasite load in spleen and liver

**Figure 2:**
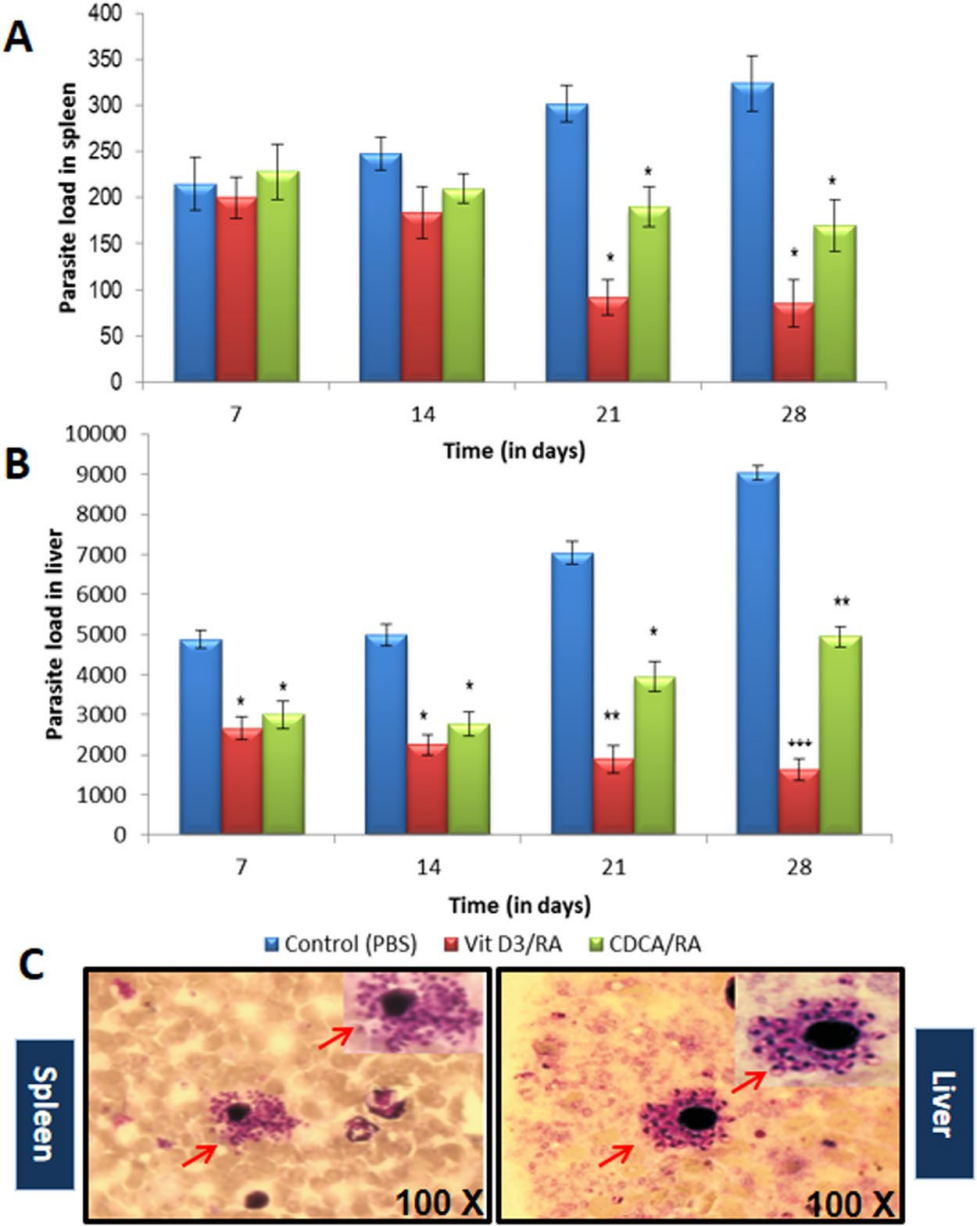
Comparison of Parasite load in spleen and liver in course of treatment with Vit.D_3_/RA, CDCA/RA *L*. *donovani* infection in BALB/c mice in comparison of control. (**A**) Parasite load in spleen (**B**) Parasite load in the liver (**C**) Giemsa stained micrograph showing macrophages (spleen and liver) infected with *L*. *donovani* showing intracellular parasite converted to amastigotes after internalization (100X; indicated by arrowheads). All the treated and control groups mice were sacrificed and Leishman-Donovan units (LDU) were calculated from liver and spleen impression smears. The values represent the mean ± SD difference between the infected and control of experiments. *P < 0.05. **P < 0.005, n = 6.

Parasite load in spleens of parasite-infected control (vehicle treated) group rose significantly. On day 7 post-treatment the mean parasite load in the control group was 215 ± 28.4 LDU. It rose to 248 ± 18 LDU on day 14, 302 ± 20.2 LDU on day 21 and 324 ± 32.3 LDU on day 28 respectively (Fig. 2A), whereas mice treated with Vit.D_3_/RA showed a significant decrease in parasite load. On day 7 PT there was little difference from the control group 200 ± 22.2 LDU (7% reduction). On day 14 P.T the parasite load in Vit.D_3_/RA treated group was 184 ± 28.3 (27%) LDU, 92 ± 19.4 (63%) LDU on day 21, and maximum reduction on day 28 PT 85 ± 25.2 (75%) LDU was observed. The group treated with CDCA/RA treatment showed a similar decrease in parasite load from 228 ± 30.4 (6%) LDU on day 7 to 210 ± 15.9 (15%) LDU on day 14, 190 ± 21.5 (37%) LDU on day 21, and maximum reduction on day 28 PT 172 ± 28.4 (47%) LDU was observed (Fig. 2A). The maximum significant reduction was seen in Vit.D_3_/RA treated group (75%) (*p* = 0.017) while in CDCA/RA treated group it was 47% (*p* = 0.045) when compared to the infected controls (Fig. 2A).

In the liver, a significant parasite load was observed in all the time periods in the vehicle treated control group as compared to the treated group. In the first week the control group showed a parasite load of 4869 ± 225 LDU. In the following weeks, parasite load decreased to 4980 ± 264 LDU on day 14 and then increased to 7036 ± 280 LDU on day 21 and 9030 ± 187 LDU on day 28 respectively (Fig. 2B). Treatment with Vit.D_3_/RA caused a significant reduction in parasite load (Fig. 2B). On day 7 PT parasite load was 2654 ± 284 LDU (45%). A reduction was observed in LDU in the following weeks. On day 14 PT, it was 2248 ± 259 LDU (50%), 1894 ± 346 LDU (73%) on day 21, and maximum reduction on day 28 PT 1640 ± 267 LDU (81%) was observed. Mice treated with CDCA/RA also displayed a significant reduction in parasite load initially, but it rose again in the final two weeks (Fig. 2B). On day 7 the LDU was 2995 ± 339 (38%), 2782 ± 300 (41%) LDU on day 14, 3948 ± 367 (44%) LDU on day 21 and 4955 ± 254 (45%) LDU on day 28 PT (Fig. 2B). Maximal reduction was seen in the treated group, i.e., Vit.D_3_/RA (81%) (*p* = 0.001) and in CDCA/RA (45%) (*p* = 0.004) on day 28 when compared to the infected controls (Fig. 2B).

The efficacy of the treatments was checked by determining the parasite load in both spleen and liver (Fig. 2C). There were significantly lower parasite loads in both spleen and liver (*p* = 0.001 to 0.0001) after treatment of the infection in all the Vit.D_3_/RA groups for oral dose and CDCA/RA molecules when compared to the vehicle treated control groups. When all the treated groups were compared, the parasite load was significantly lower in the liver for the Vit.D_3_/RA treated group as compared to the CDCA/RA treated group. Similarly, the parasite load in spleen showed the maximum reduction in the Vit.D_3_/RA treated group, as compared to CDCA/RA treated group.

### Effect of drug treatment on immune response (Th1 and Th2)

**Figure 3:**
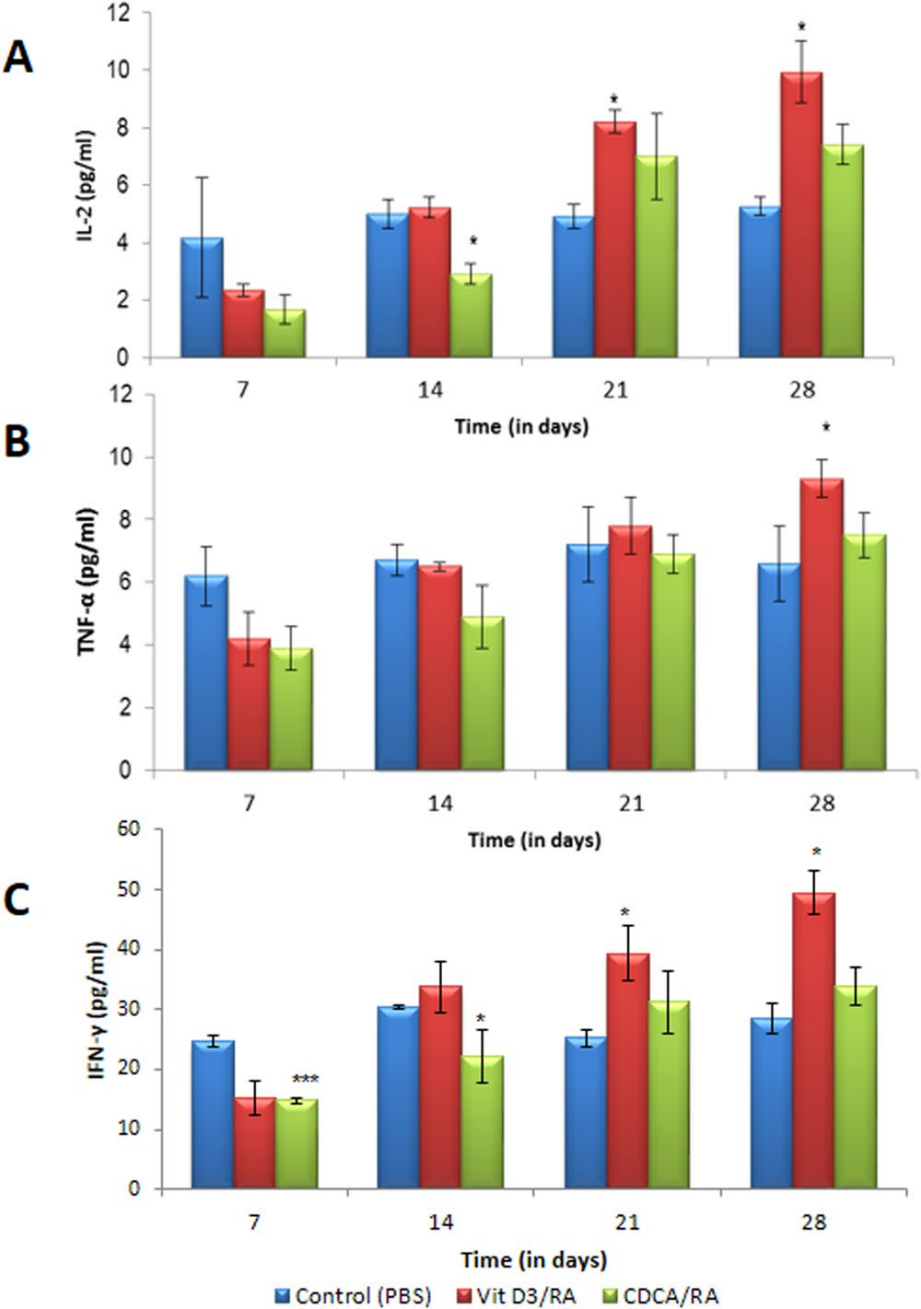
Comparison of Th1 serum cytokine levels secreted by *L*. *donovani* infected BALB/c mice treated with Vit.D_3_/RA and CDCA/RA at different time intervals. (**A**) IL-2 (**B**) TNF-α (**C**) IFN-γ. The values represent the mean ± SD difference between the infected and control groups. *P < 0.05. **P < 0.005, ***P < 0.0001, n = 6.

**Figure 4:**
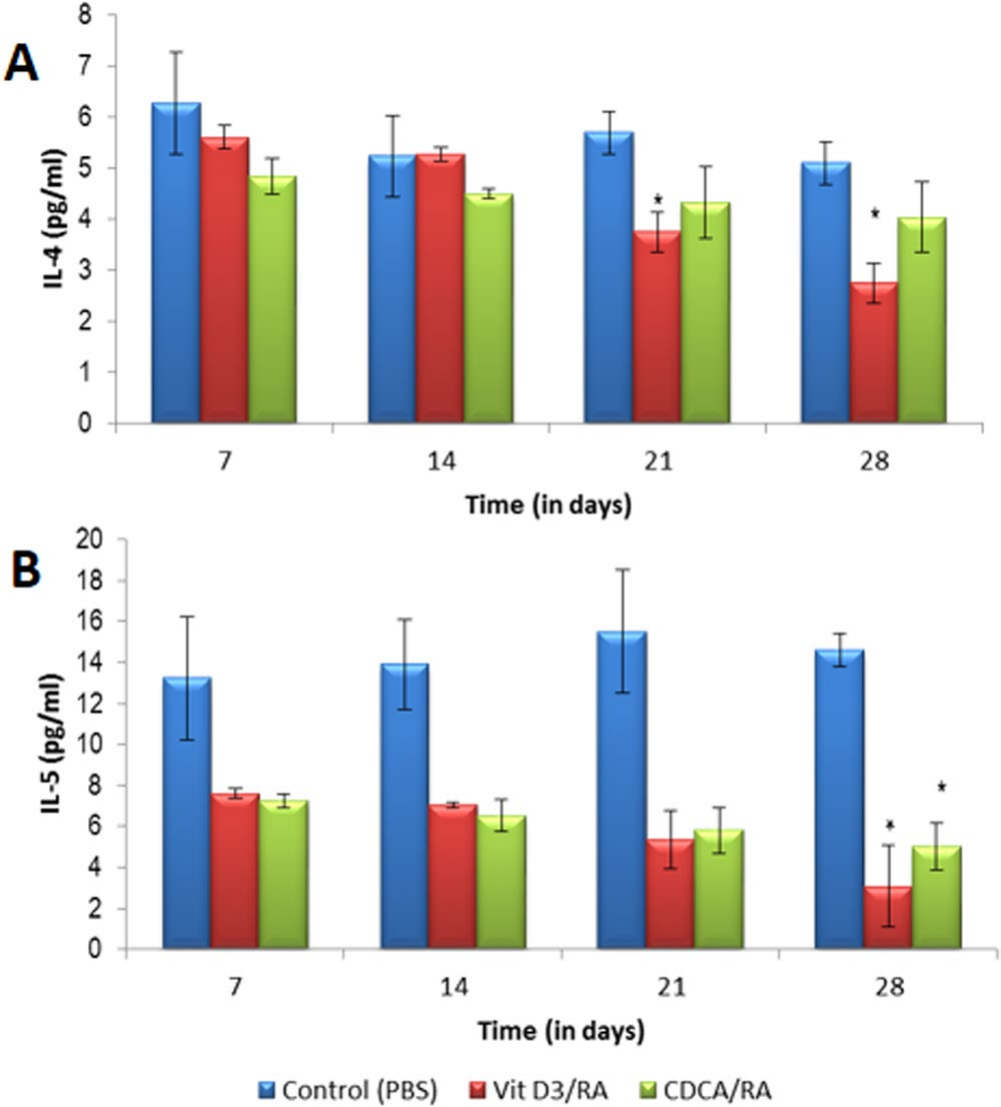
The effect of Th2 serum cytokine levels secreted by *L*. *donovani* infected BALB/c mice treated with Vit.D_3_/RA and CDCA/RA at different time intervals. (**A**) IL-4 (**B**) IL-5. The values represent the mean ± SD difference between the infected and control groups. *P < 0.05. **P < 0.001, n = 6.

The production of Th1 (IL-2, TNF-α, IFN-γ) and Th2 (IL-4 and IL-5) cytokines in BALB/c mice (serum samples) infected with *L*. *donovani* and treated with the molecules Vit.D_3_/RA and CDCA/RA was assessed. Oral Vit.D_3_/RA and CDCA/RA treated mice showed IL-2 response on day 21 PT (8.2 ± 0.39 and 7.0 ± 1.5 respectively), and these levels rose significantly on day 28 to 9.92 ± 1.08 and 7.45 ± 0.7 respectively (Fig. 3A) as compared to control group after 4 weeks of infection. In both drug-treated groups (Vit.D_3_/RA, CDCA/RA), TNF-α, a cytokine which is a marker of the Th1 type of immune response, remained at basal levels on all PT day. Maximal observed levels of TNF-α were 9.3 ± 0.6 pg/ml for Vit.D_3_/RA and 7.7 ± 0.71 for CDCA/RA on day 28 PT (Fig. 3B). There was no significant difference in TNF-α levels between control and treatment groups (*p* > 0.05) except on day 28 PT in Vit.D_3_/RA group (*p* = 0.038). There was a significant upregulation of IFN-γ levels in the serum of mice treated with Vit.D_3_/RA on days 21 and 28 PT when compared to vehicle treated control mice. The highest level of IFN-γ levels were observed on day 28 PT, which was 49.47 ± 3.6 pg/ml. In the CDCA/RA group, IFN-γ levels were not significantly different from the control group (*p* > 0.05); the maximal levels on day 28 PT were 33.9 ± 3.1 pg/ml (Fig. 3C). The maximum level of this cytokine in the vehicle treated control group was 30.4 ± 0.9 pg/ml. There was no significant difference in IL-4 levels of various post-treatment days in control (vehicle treated) group. In the Vit.D_3_/RA treated group, no significant difference was found in the levels of IL-4 on day 7 and 14 PT, but a significant decrease was observed on days 21 and 28, i.e., 3.75 ± 0.38 & 2.75 ± 0.41 pg/ml (Fig. 4A). In the CDCA/RA treated group, no significant decline in the level of IL-4 response was found on days 21 or 28 PT (4.33 ± 5.2 pg/ml and 4.04 ± 3.18 pg/ml respectively) (Fig. 4A). IL-5 is another marker of the Th2 type of immune response. In the control group, levels of IL-5 were not significantly different on various days PI. In both treated groups (Vit.D_3_/RA and CDCA/RA), on all days PT, the levels of IL-5 were lower than the control group, but the difference was not statistically significant except on day 28 PT (3.08 ± 2.3 pg/ml and 4.9 ± 1.16 pg/ml respectively) (Fig. 4B).

### Effect of drug treatment on Histopathological responses

**Figure 5:**
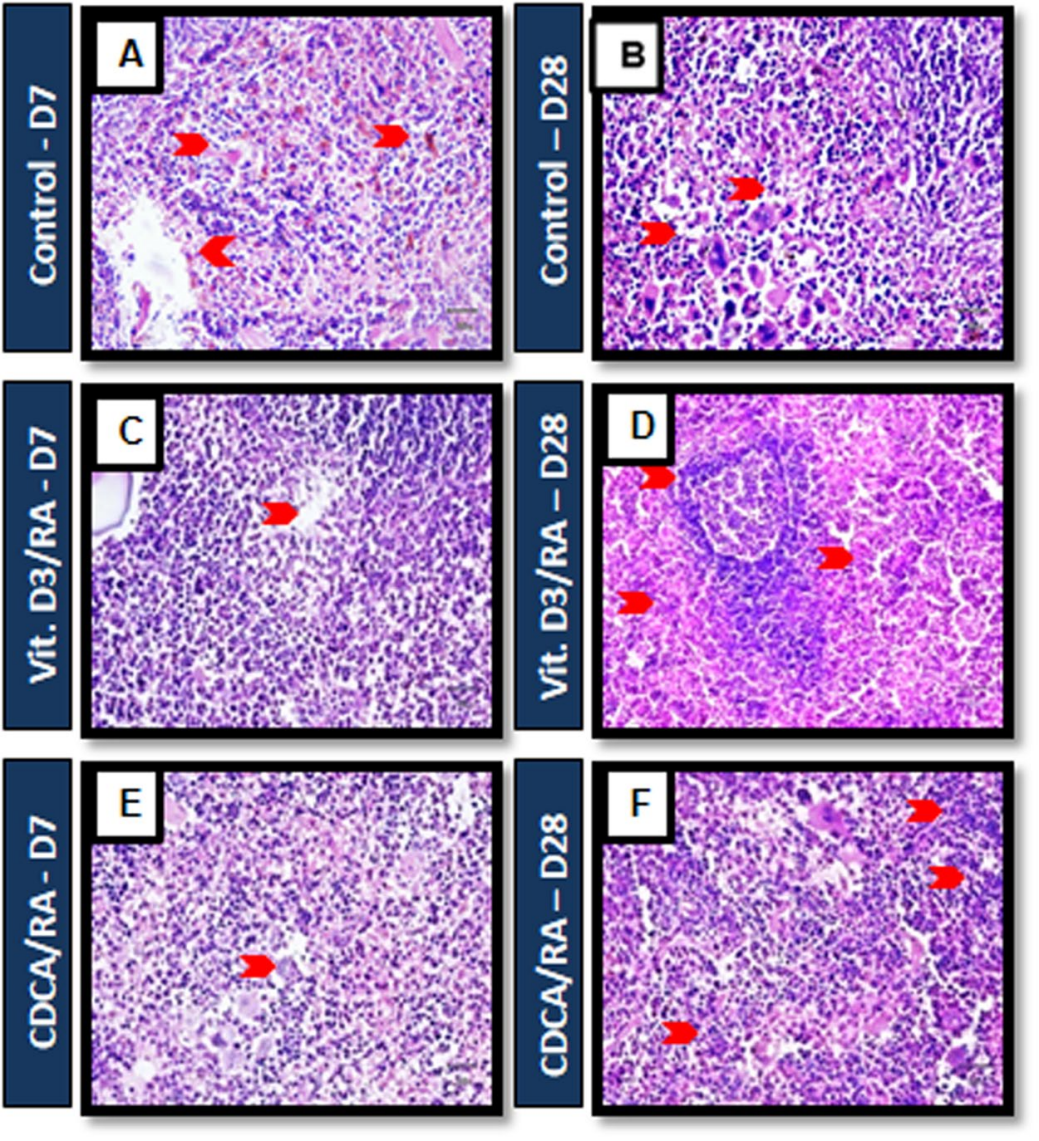
Mouse infected with *L*. *donovani* splenic tissue histological staining of H and E. (**A**,**B**) Control mice Day 7 and Day 28: *L*. *donovani* infected mice showed reactive follicular hyperplasia, including expansion of marginal zone around the follicle and immature granuloma (**C**,**E**) mice treated with Vit.D_3_/RA and CDCA/RA displayed reactive hyperplasia of lymphoid follicles and early development of granuloma in the red pulp by day 7 of the PT. (**D**) 28 PT, mice treated with Vit.D_3_/RA showed effective response through the recruitment of red pulp with small lymphoid follicles and absence of granuloma formation. Similarly, mice treated with CDCA/RA (**F**) showed shrinkage in white pulp and the development of red pulp with no granuloma (arrow head, respectively), n = 6.

Histology of the spleen sections from *L*. *donovani* infected mice showed enlarged reactive follicular hyperplasia, including expansion of the marginal zone around the follicle, and immature granuloma developed by day 7 in infected controls (Fig. 5A,B). On day 28, we observed the expansion of white pulp without hyperplasia with a few lymphoid follicles in diffuse lymphoid cells and occasional granuloma. However, mice treated with Vit.D_3_/RA and CDCA/RA showed reactive hyperplasia of lymphoid follicles and early development of granuloma in the red pulp by day 7 of the PT (Fig. 5C,E). On day 28 PT, mice treated with Vit.D_3_/RA showed effective response through the recruitment of red pulp in a wide area with small lymphoid follicles and absence of granuloma formation with a cluster of pale histiocytes and lymphocytes (Fig. 5D). Likewise, mice treated with CDCA/RA displayed shrinkage in white pulp and the growth of red pulp with lymphoid follicles and rising granuloma (Fig. 5F).

## Discussion

In this present study, we studied the Vitamins (A&D) and Isoprenoid (Chenodeoxycholic acid) molecules antileishmanial role through Th1 immunostimulatory responses and their effect on parasite burden. To date, treatment regimens for lesihmanisis patients are dependent on the FDA-approved oral agent miltefosine, parental agents such as amphotericin B deoxycholate and pentamidine isethionate, as well as azoles. Nevertheless, many challenges remain, which include efficacy, availability of drugs, cost of treatment (drugs and hospitalization), adverse effects, and rising parasite resistance. Our previous *in vitro* study^9^ indicated that Vit.D_3_, RA, and CDCA combination can modulate the macrophage infected parasite burden. For this reason, we have made an attempt to understand the important role of vitamins and isoprenoids combination effect on host immune modulation and *L*. *donovani* infection dynamics in a mouse model (*in-vivo*).

The BALB/c mouse model has been used widely in the visceral form of leishmaniasis in relation to pathology, immunology and drug discovery^20,21^. In earlier studies, various inbred strains of mice following *L*. *donovani* infection have demonstrated that liver and spleen are the principle organs affected and an increase in their weight was observed^20^. The parasite load in the liver and spleens of the mice at various PI gave a better index of infectivity in terms of Leishmania-Donovanon units (LDU)^20,21^. In this study, we found that drug treatments were effective in reducing parasite burden after its establishment, in comparison with vehicle treated control animals. Interestingly, the mice treated with Vit.D_3_/RA showed a significant reduction in spleen and liver weights, 77% and 75% respectively (Fig. 1A,B). In addition, a reduction in parasite load was observed, i.e., 75% LDU in the spleen and 81% LDU in the liver compared to a vehicle treated control group (Fig. 2A). For the CDCA/RA group, significant weight reduction was noticed in spleen (64%) and liver (46%) with reductions of LDU (47% and 45% respectively) in comparison to control group (Figs 1 and 2).

Further, we examined the serum Th1 & Th2 cytokine levels of treated and control mice at different time points of treatment by flow cytometry. The cytokine response after infection of *L*. *donovani* over the course of treatment with the Vit.D_3_/RA/CDCA combination using a CBA cytokine assay showed that relative induction of Th1 and Th2 cytokine response varies with time. In rodent models, the Th1/Th2 paradigm is important in determining the outcome of murine L. major infection^22^. In the present study, we observed that mice infected with *L*. *donovani* had an activation of Th1 cell response to treatment with Vit.D_3_/RA and CDCA/RA (Fig. 3). Mice treated with Vit.D_3_/RA displayed a gradual increase of all Th1 cytokines, which at the end of 4th week (i.e., day 28) PT was significant. Interestingly, the CDCA/RA group also showed a slower rate of diversion towards Th1 immune response at the end of treatment as compared to the vehicle treated control group (Fig. 3), which correlated with the reduction of parasite load and weights of spleen and liver. IFN-γ plays a key role in the control of infection by many intracellular pathogens, including Leishmania spp., and is primarily responsible for macrophage activation and the killing of intracellular parasites^23^. Protective immunity against VL, as in case of cutaneous leishmaniasis is dependent on IL-12 driven type 1 response characterized by IL-2 and IFN-γ production, which induces parasite death^24^. Hakim *et al*.^25^ suggested that 1, 25 (OH) 2 D3 increases TNF-α transcript abundance in bone marrow macrophages via a transcriptional mechanism. IFN-γ is essential for the development and propagation of Th1 type immune response^26^ and is a potent activator of monocytic cell functions, including the stimulation of TNF-α and control of IL-6 synthesis. Monick *et al*.^27^ showed that TNF-α strengthens the Th1 response. It is possible that the association of Th1 immune modulatory effects could have contributed by increasing the production of nitric oxide induced by TNF-α, favoring the elimination of the parasite^28^ and controlling the inflammatory response, minimizing tissue damage and prolonging the survival of BALB/c mice. In contrast, Th2 cytokine IL-4 response has no difference during the first two weeks of Vit.D_3_/RA treatment. A gradual decrease was observed over the next two weeks in treating animals, which was found to be statistically significant (Fig. 4A). Likewise, another Th2 cytokine IL-5 showed a significantly reduced only on day 28 PT. In the CDCA/RA group, a similar trend in IL-5 levels was observed, but there was no significant decline in levels of IL-4 (Fig. 4). Regarding immune responses in human acute VL, the cytokine profile shows high production of IL-4 and IL-10 and low IL-2, and IFN-γ production^29^. A range of innate and environmental factors^30^ and effector cells (natural killer cells, CD8+ T cells, and neutrophils) modulate the immune response in visceral infection^31^.

Histologically, an increased recruitment of granuloma was induced by *L*. *donovani* in BALB/c, which may contribute to the development of a Th2 response in the early stages of infection. Mice treated with Vit.D_3_/RA and CDCA/RA may contribute to an effective response (Vit.D_3_/RA being the most effective) against *L*. *donovani* through the recruitment of inflammatory cells in early infection, as well as Th1 cells in advanced stages of infection, resulting in absence of granuloma (Fig. 5A,B). All animals in the Vit.D_3_/RA and CDCA/RA groups demonstrated mature granulomas in the spleen (Fig. 5D,F), which might be responsible for the significant decrease in parasite burden in the spleen.

This study evaluated the effect of immunomodulatory drugs on the outcome of infection by *L*. *donovani* in animal models. As with our previous *in vitro* study, the use of a combination of Vit.D_3_/RA/CDCA revealed the downregulation of TACO mRNA expression and the significant inhibition of parasite load as compared to vehicle treated control groups^9^. Notably, our *in vitro* data linked that level of TACO transcription to the survival or killing/inhibition of *L*. *donovani*. Recent studies revealed important information about the Vit.D role in the regulation of leishmaniasis^32–34^. In addition to Vit. D, the role of Vit. A in immune responses in leishmaniasis is also studied^15^. Although these studies were not investigating the specific Th1 and Th2 immune responses. In this present study, we found that a vitamin and Isoprenoid combination was highly potent in restricting parasite load *in-vivo* with immune modulation during treatment in mouse models. This approach seems more convincing, keeping in view the existing hurdles with currently available drugs for leishmaniasis. Vitamins and isoprenoid molecules are cost-effective to produce, distribute and deliver to humans, and can be orally administered. Therefore, it may be a valuable and rational candidate for combination therapy in areas where leishmaniasis is endemic.

**Figure 6:**
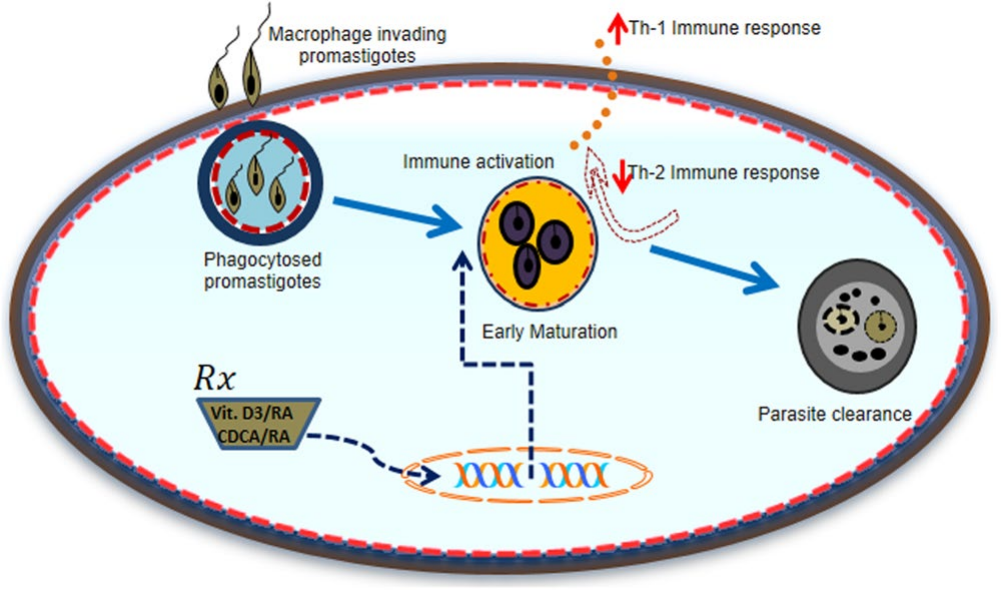
Working model  describes the pathways potentially involved in host immune modulation  against Leishmania infection and parasite clearance with Vit.D_3_/RA/CDCA molecules. Leishmania promastigotes binding to macrophages enter by phagocytosis. Vit.D_3_/RA and CDCA/RA treatment effects on the early maturation of phagosomes by immune modulation towards Th1 immune response and downregulation of Th2 immune response leads to parasite clearance.

In conclusion, there is a lack of research to address the role of vitamins and isoprenoids in regulation of leishmania infection. This study reinforces the importance of vitamin A (RA), vitamin D (Vit. D_3_) and CDCA combination in effectively controlling parasite growth and infectivity. The research strategy of this study was to stimulate the beneficial immune responses and at the same time to inhibit harmful immune activity in the host. Our findings suggest that treatment with the above molecules has a significant reduction of parasite load, modulated pro-inflammatory (Th2) immune response to anti-inflammatory (Th1) immune activation (Fig. 6). Further, the resulting formulation was found to be effective in combination therapy by the oral route in a mouse model of VL. Such combinations could be effective, less toxic, and affordable, with good patient compliance, compared to the current alternatives. Taken together, our data expands understanding of the combined therapy of the anti-leishmanial role of Vit.D_3_, RA and CDCA, which is apparently potentiated by their immunostimulatory capacity and may be beneficial in the treatment of VL.
